# Exosomal microRNA remodels the tumor microenvironment

**DOI:** 10.7717/peerj.4196

**Published:** 2017-12-22

**Authors:** Xiaoli Jiang, Song Hu, Qiang Liu, Caiyun Qian, Zhuoqi Liu, Daya Luo

**Affiliations:** 1 Department of Biochemistry and Molecular Biology, School of Basic Medical Sciences, Nanchang University, Nanchang, People’s Republic of China; 2 Queen Mary School, School of Medicine, Nanchang University, Nanchang, People’s Republic of China; 3 First Clinical Medical College, School of Medicine, Nanchang University, Nanchang, People’s Republic of China; 4 Jiangxi Province Key Laboratory of Tumor Pathogens and Molecular Pathology, Nanchang University, Nanchang, People’s Republic of China

**Keywords:** Endogenous, Exogenous, Exosome, microRNA, Tumor microenvironment

## Abstract

Tumor occurrence, progression and metastasis depend on the crosstalk between tumor cells and stromal cells and on extrinsic factors outside the tumor microenvironment. Exosomal microRNA (miRNA) not only is involved in communications within the tumor microenvironment but also mediates communications between the extrinsic environment and tumor microenvironment. However, most reviews have been limited to the role of endogenous exosomal miRNA in remodeling the tumor microenvironment. Hence, we herein review the role of endogenous exosomal miRNA in mediating intercellular crosstalk within the tumor microenvironment, inducing the formation of the premetastatic niche. To place our vision outside the microenvironment, we also summarize for the first time the most recent studies regarding how exogenous miRNA derived from milk, plants and microbes influences the tumor microenvironment. Furthermore, to improve the value of exosomal miRNA in cancer research and clinical applications, we also provide some novel ideas for future research based on the comprehensive role of exosomal miRNA in remodeling the tumor microenvironment.

## Introduction

Researchers are gaining an improved understanding of tumor evolution, and the role of the tumor microenvironment in tumor occurrence and development has accordingly attracted increasing attention. The tumor microenvironment is composed of various types of cells, including tumor cells and a variety of stromal cells, such as endothelial cells, immune cells, fibroblasts, adipocytes and mesenchymal stem cells. The occurrence, development and metastasis of a tumor depend not only on its own characteristics but also on crosstalk between tumor cells and stromal cells in the tumor microenvironment (Hanahan & Weinberg, 2011). In normal tissues, stromal cells inhibit tumorigenesis; however, such communication in the malignancy-transformed tumor microenvironment promotes tumor development (Junttila & de Sauvage, 2013).

**Figure 1:**
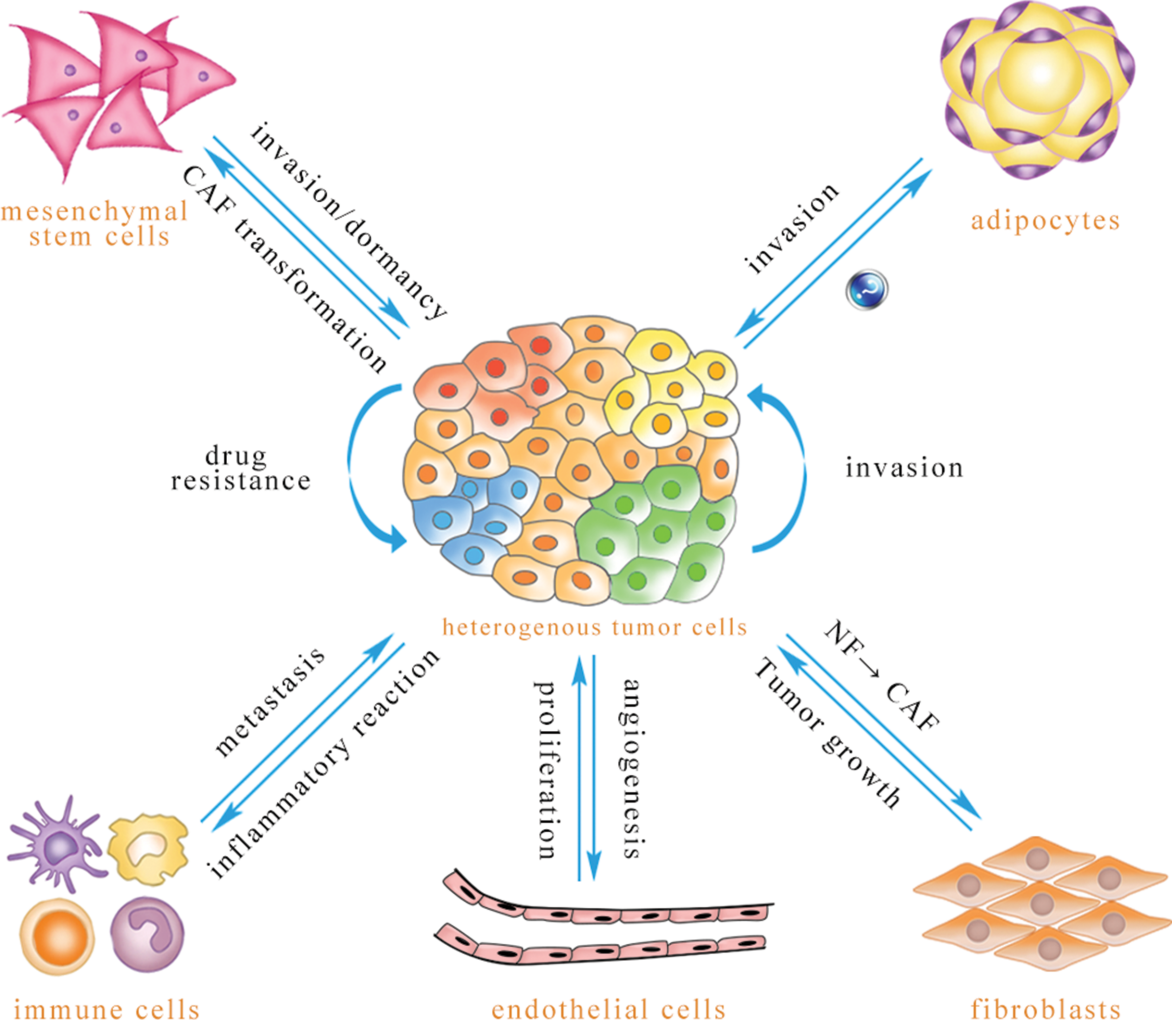
Exosomal miRNA-mediated intercellular cross-talk within the tumor microenvironment. Through intercellular transfer of exosomal miRNA, tumor parenchymal cells can confer drug resistance to each other and enhance the invasiveness of recipient cells. Tumor cells can promote angiogenesis of endothelial cells, and endothelial cells can promote tumor cell proliferation. Immune cells are able to regulate tumor metastasis under different conditions, and tumor cells may induce immune cell dysfunction and pro-inflammatory cytokine release. Tumor cells are capable of inducing CAF phenotype transformation of mesenchymal stem cells, and mesenchymal stem cells can be transferred to the tumor site to promote tumor metastasis or dormancy, but they also inhibit tumor growth in some cases. Adipocytes play an important role in promoting tumor cell invasion, while the effect of tumor-secreted exosomes on adipocytes has not been reported to date. Exosomal miRNA can convert normal fibroblasts into CAFs for tumor survival, and CAFs can promote tumor progression (The direction of exosomal miRNA transfer is denoted by blue arrows; the annotations which are close to the arrows refer to biological effects that are regulated in recipient cells).

microRNA (miRNA) comprises a class of noncoding RNA of 21–24 nucleotides (Li et al., 2010). A mature miRNA, which is derived from its primary miRNA transcript via RNase cleavage, has the ability to negatively regulate the expression of protein-coding genes through formation of the RNA-induced silencing complex (RISC) (Xing et al., 2014). miRNA is involved in many important biological functions, including cell proliferation, differentiation, apoptosis, metabolism and drug resistance (Ambros, 2003; Calin & Croce, 2006). miRNA is present in cells and can also be released into the extracellular environment through a variety of routes, including exosome. The diameter of an exosome is approximately 30–100 nm, with a density of 1.13–1.19 g/ml. These vesicles are secreted by various cells, including tumor cells, endothelial cells, immune cells, fibroblasts, adipocytes, and mesenchymal stem cells (Neviani & Fabbri, 2015; Ohshima et al., 2010; Singh et al., 2014). As intercellular signaling molecules in the tumor microenvironment, exosomes contain many biological components: RNAs, miRNAs, proteins, lipids, and many small metabolites. Exosomes are absorbed by receptor cells through membrane fusion, endocytosis and ligand–receptor binding (Bovy et al., 2015; Fabbri et al., 2012; Montecalvo et al., 2012), resulting in biological changes in these recipient cells (Fig. 1).

Because exosomes can be detected in almost all bodily fluids and because these vesicles are easily absorbed by recipient cells due to their nanoscale structures, exosomes may be valuable for early diagnosis, curative effect assessment, prognostic analysis and drug target development (Armstrong et al., 2015; Lai et al., 2013; Zhou et al., 2014b). This review summarizes how endogenous and exogenous exosomal miRNAs mediate crosstalk between tumor cells and stromal cells to reprogram the tumor microenvironment.

## Survey Methodology

PubMed was mainly used to search for related articles published using the keyword “exosome,” “miRNA” and “cancer.” Then, screened articles were used as references for this review. Additional keywords, such as “exogenous,” “virus” and “bacteria,” were also used.

## Endogenous Exosomal miRNA Mediates Intercellular Crosstalk in the Tumor Microenvironment

Various cell-derived endogenous exosomes function via the paracrine or autocrine signaling pathway. The endogenous exosomal miRNA-regulated network connects many types of cells in the microenvironment that can act as the donor or recipient of exosomal miRNA. Through intercellular transfer of exosomal miRNA, tumor parenchymal cells can “educate” stromal cells, and stromal cells in turn regulate the proliferation, apoptosis, migration and invasiveness of tumor cells. A mutual loop may also form between tumor parenchymal and stromal cells.

### Crosstalk among tumor cells

Tumor cells modulate the biological behavior of nearby cells via paracrine and autocrine signaling. Exosomes released from breast cancer MCF-7 cells can be ingested by other MCF-7 cells and promote tumor proliferation in the recipient cells by downregulating expression of the pro-apoptotic protein Bax. In turn, shikonin (SK) inhibits proliferation of MCF-7 breast cancer cells by suppressing exosome release, thus exerting an anticancer effect (Wei et al., 2016).

Different malignant tumor cells can affect the invasion and migration of tumor cells by releasing exosomal miRNA. Malignant MDA-MB-231 breast cancer cells contain a large abundance of miR-10b, which can be released to the extracellular environment via exosomes and transferred to nonmalignant HMLE breast cells to suppress expression of homeobox D10 (HOXD10) and Kruppel-like factor 4 (KLF4), thereby promoting HMLE cell invasion and migration (Singh et al., 2014).

Tumors comprise a class of highly heterogeneous diseases. Indeed, a specific tumor is composed of groups of clonal cells that have different growth rates and different sensitivities to chemotherapy and radiotherapy. Thus, tumor cells with different malignancies have different abilities to metastasize and invade. An overall understanding of the heterogeneity of tumor cells and communication among heterogeneous tumor cells via miRNA transfer may help guide clinical treatments.

A Cy3-labeled miR-21 mimic harbored by the human esophageal cancer EC9706 cells were found to be transferred to untransfected EC9706 cells via exosomes, and this exosomal miR-21 directly suppressed its target, programmed cell death protein 4 (PDCD4), and activated the JNK signaling pathway to promote migration and invasion of the recipient cells (Liao et al., 2016). Similarly, a hypoxic tumor microenvironment can induce oral squamous cell carcinoma cells (OSCCs) to generate more miR-21-containing exosomes that are then transferred to normoxic OSCCs to enhance their migration and invasion by inhibiting expression of phosphatase and tensin homolog (PTEN) and PDCD4 (Li et al., 2016). Tumor cells can also confer drug resistance via exosomal miRNA. For example, Mao et al. reported that adriamycin-sensitive breast cancer cells can acquire drug resistance after internalizing exosomes derived from adriamycin-resistant breast cancer cells. The underlying mechanism involved inhibition of Sprouty 2, p27 and PTEN expression in adriamycin-sensitive cells by miR-23a, miR-24 and miR-222, respectively, within the transferred exosomes (Mao et al., 2016).

### Tumor cells and endothelial cells

It is well known that tumor cells have a high demand for energy and that a suitable blood supply can help tumor cells acquire oxygen and nutrients and remove tumor cell metabolites, which are closely related to the function of endothelial cells in the tumor microenvironment. The interaction between tumor parenchymal cells and endothelial cells mediated by exosomal miRNA can affect tumor progression.

In vitro, human leukemia K562 cells secrete a large number of miR-17/92 clusters (especially miR-92a) into the culture supernatant. These miRNAs can then be transferred to endothelial cells via exosomes, promoting angiogenesis and endothelial cell migration (Umezu et al., 2013). Umezu et al. (2014) showed that hypoxia-resistant multiple myeloma (HR-MM) cells, which were screened under a condition of sustained hypoxia for six months, released more exosomal miR-135b, which promoted angiogenesis through the hypoxia-inducible factor–factor inhibiting hypoxia-inducible factor 1 (HIF–FIH) signaling pathway in endothelial cells, than the parent cells. Similarly, culturing the human leukemia K562 cell line under hypoxic conditions for 24 h resulted in significantly increased levels of exosomal miR-210 and significantly promoted angiogenesis of endothelial cells in a co-culture system (Tadokoro et al., 2013). Furthermore, exosomal miRNA mediated the anti-angiogenesis effect of the natural anticancer compound docosahexaenoic acid (DHA). In fact, DHA-treated breast cancer cells exhibit significantly enhanced secretion of exosomes and increased levels of let-7a, miR-21, miR-23b, miR-27b and miR-320b within these exosomes. The exosomal miRNAs are transferred to endothelial cells to directly suppress pro-angiogenic target genes, such as plasminogen activator and angiomotin-like 1, inhibiting angiogenesis. This finding indicates that exosomal miRNAs are capable of mediating the anti-angiogenic action of DHA (Hannafon et al., 2015).

In addition, endothelial cell-derived exosomal miRNA also regulates the biological behavior of tumor parenchymal cells. By adding vascular endothelial growth factor (VEGF) and basic fibroblast growth factor to mimic the tumor microenvironment in the culture medium of endothelial cells, exosome release and the level of miR-503 in exosomes was reduced, resulting in decreased transfer of endothelial cell-derived exosomal miR-503 to the tumor cells. Decreased miR-503 resulted in upregulation of cyclin D2 and cyclin D3 to promote tumor cell proliferation. However, an elevated plasma level of miR-503 was observed after neoadjuvant chemotherapy, which may be explained in part by enhanced endothelial cell secretion. This process may augment the efficacy of chemotherapy drugs (Bovy et al., 2015).

### Tumor cells and immune cells

The immune microenvironment of the host is closely related to the occurrence and development of tumors. The large numbers of infiltrating immune cells in tumor tissues are among the important components of the tumor microenvironment (Cook & Hagemann, 2013). The immune system exerts two opposing functions at different stages of tumor development. For example, the immune system can recognize tumor antigens and play a positive role in tumoricidal activity at the initial stage of tumorigenesis by releasing cytokines. With tumor development, tumor cells not only evade the immune system but also stimulate immune cells to release more tumor growth factors (such as transforming growth factor beta (TGF-β) to support tumor growth. As an intercellular messenger within the tumor microenvironment, exosomal miRNA also participates in this process.

Dendritic cells are professional antigen-presenting cells that efficiently ingest, process and present antigens, playing an important role in the initiation, regulation and maintenance of the immune response (Reis e Sousa, 2006). Compared with normal pancreatic tissue, miR-203 overexpressed by pancreatic cancer cells can be transferred to dendritic cells via exosomes, resulting in dendritic cell dysfunction via downregulation of Toll-like receptor 4 (TLR4) expression and its downstream genes, such as tumor necrosis factor alpha (TNF-α) and interleukin 12 (IL-12) (Zhou et al., 2014a). Fabbri et al. first showed that exosomal miRNA directly activates TLRs. Liver cancer cells can secrete large numbers of miR-21- and miR-29a-containing exosomes, and these miRNAs function as ligands of TLRs on immune cells. This process results in the release of IL-6, TNF-α and other pro-inflammatory cytokines through a nuclear factor kappa B (NF-κB) pathway-mediated pro-inflammatory response, thereby shaping the tumor microenvironment into a pro-inflammatory and pro-metastatic niche (Fabbri et al., 2012). Compared with normal oxygen conditions, hypoxia does not promote the release of exosomes from nasopharyngeal carcinoma cells. However, the miR-24-3p content in exosomes increases significantly, which can downregulate fibroblast growth factor 11 (FGF11) protein expression in T cells. This decreased expression of the FGF11 protein inhibits the differentiation of T cells toward Th1 and Th17 cells by inhibiting signal transducer and activator of transcription 1 (STAT1) and signal transducer and activator of transcription 3 (STAT3) protein phosphorylation while enhancing STAT5 protein phosphorylation to promote recruitment of Treg cells, which help tumor cells evade the immune system (Ye et al., 2014, 2016). In addition to the exosomal miRNA-mediated tumor-promoting function mentioned above, exosomal miRNA also plays a role in tumor suppression. Ji-Young Jang reported that epigallocatechin gallate-treated murine breast cancer 4T1 cells showed elevated levels of miR-16, which can be taken up by tumor-associated macrophages (TAMs) via exosomes to reduce TAM infiltration and M2 polarization, thus inhibiting tumor growth (Jang et al., 2013).

At the same time, exosomal miRNA secreted by immune cells can be transferred to tumor cells. Hu et al. found that the level and release of exosomal miR-7 was increased after stimulating macrophages with TNF-like weak inducer of apoptosis (TWEAK). Exosomes can also be transferred to epithelial ovarian cancer (EOC) cells, and miR-7 can inhibit the EGFR/AKT/ERK1/2 signaling pathway to block the metastasis of EOC (Hu et al., 2017). Similarly, exosomes released by IL-4-activated macrophages, which specifically contain miR-233, can be transferred to breast cancer cells and promote their invasiveness by regulating the myocyte enhancer factor 2c (Mef2c)-β-catenin signaling pathway (Yang et al., 2011).

A mutual loop mediated by exosomal miRNA may also form between tumor cells and immune cells to promote tumor development. Exosomal miR-21 released by neuroblastoma cells (NBLs) can be ingested by mononuclear cells to elicit NF-κB signaling pathway and can induce mononuclear cells to prevalently differentiate into M2 macrophages. miR-155 in turn released by mononuclear cells can be taken up by neuroblastoma cells, reducing expression of the telomerase inhibitor telomeric repeat-binding factor 1 (TERF1), enhancing the activity of telomerase and promoting drug resistance in tumor cells (Challagundla et al., 2015).

### Tumor cells and fibroblasts

Fibroblasts are the major cellular components of the tissue matrix and have the ability to synthesize and secrete extracellular matrix (ECM) proteins and regulate tumor angiogenesis, epithelial–mesenchymal transition (EMT), and tumor metastasis (Kohlhapp et al., 2015). Normal fibroblasts (NFs) inhibit tumor growth, whereas cancer-associated fibroblasts (CAFs) promote tumor progression (Bremnes et al., 2011; Parrott et al., 2001). Exosomal miRNA can convert normal fibroblasts into CAFs for tumor survival.

Pang et al. found that NFs can be transformed into CAFs or cells with a CAF phenotype by co-culturing NFs with pancreatic cancer cells or by treating NFs with pancreatic cancer cell-derived microvesicles (MVs). One possible mechanism for this transformation is that pancreatic cancer cells are able to secrete miR-155-containing exosomes, which can be received by NFs and convert them into CAFs via inhibition of tumor protein p53-inducible nuclear protein 1 (TP53INP1), a target protein of miR-155 (Pang et al., 2015). Another group has reported that miR-409 is highly expressed in CAFs isolated from patient samples by laser capture microscopy. Overexpression of miR-409 in normal fibroblasts can transform them into CAFs. The miRNA can be transferred to prostate cancer cells via exosomes and inhibit expression of tumor-suppressor genes, such as Ras inhibitor 1 and matrix antigen 2, and enhance tumor growth, promote EMT in tumor cells and maintain the characteristics of stem cells (Josson et al., 2015). Zhang et al. analyzed sequences of miRNAs in exosomes secreted from CAFs and corresponding paracancer fibroblasts from hepatocellular carcinoma patients, and the abundance of miR-320a was found to be significantly decreased in these CAF exosomes. As an anticancer miRNA, miR-320a inhibits mitogen-activated protein kinase (MAPK) signaling by reducing expression of the proto-oncogene pre-B-cell leukemia transcription factor 3 (PBX3). Therefore, decreased expression of miR-320a in CAF-derived exosomes contributes to the aggressive phenotype transition of hepatocellular carcinoma (HCC) (Zhang et al., 2017).

Cancer-associated fibroblasts can help pancreatic cancer cells develop drug resistance to gemcitabine, the main chemotherapeutic drug for pancreatic ductal adenocarcinoma. Gemcitabine treatment not only promotes the release of CAF exosomes but also increases expression of the chemotherapy resistance factor snail and its target miR-146a in exosomes. After exosomes are absorbed by pancreatic cancer epithelial cells, the levels of the transcription factor snail and miR-146a are increased, promoting the resistance of these tumor cells to gemcitabine (Richards et al., 2017).

### Tumor cells and adipocytes

As a common social phenomenon worldwide, obesity is known to be associated with certain chronic diseases, but it is also one of the major risk factors for cancer, and it may indicate a bad prognosis in several types of cancer (Renehan, Zwahlen & Egger, 2015). Adipocytes are among the major components of adipose tissue, which can secrete a variety of adipokines and provide energy for tumor cells (Iyengar et al., 2003; Nieman et al., 2011). Because of stress conditions that exist in obesity, such as inflammation and hypoxia, adipocytes can release more exosomes (Clement et al., 2017), and mounting evidence has shown that the exosomal miRNAs secreted by adipocytes are also involved in the occurrence and development of tumors.

For instance, Au Yeung and other researchers found that tumor-associated adipocytes express a high abundance of miR-21, which can be transferred to ovarian cancer cells via exosomes to promote their motility and invasiveness. Exosomal miR-21 can directly downregulate the expression of apoptotic protease activating factor 1 (APAF1), a tumor drug resistance and apoptosis-associated protein that promotes drug resistance and aggressiveness in ovarian cancer cells (Au Yeung et al., 2016). Mouse preadipocytes (3T3L1) treated with the natural antitumor compound SK release exosomes with high levels of miR-140. Ingestion of exosome miR-140 by the early noninvasive breast cancer MCF10DCIS cell line inhibits the stemness of the tumor cells by downregulating the transcription factor SOX9/SOX2. Exosomes secreted by SK-treated preadipocytes significantly reduce secretion of exosomal cytokines, many of which are involved in tumor growth, metastasis and angiogenesis. These findings suggest that targeting preadipocytes in the tumor microenvironment may interfere with tumor development (Gernapudi et al., 2015).

Regardless, the effect of tumor-secreted exosomes on adipocytes has not been reported to date. We look forward to more studies exploring this topic to contribute to our understanding of the crosstalk between tumor cells and adipocytes.

### Tumor cells and mesenchymal stem cells

Mesenchymal stem cells (MSCs) are collectively derived from the mesoderm and have the ability to migrate to specific tissues, a process also known as homing. MSCs show a double-edged sword effect on tumor development: MSCs can be transferred to the tumor site to promote tumorigenesis and tumor metastasis, but they also inhibit tumor growth. This dual effect may be related to the source of MSCs, the type of tumor cells and the distribution of cytokines in the microenvironment (Reza et al., 2016). Similarly, exosomal miRNA performs a regulatory role in the process of MSC-mediated tumor development.

Mesenchymal stem cells take up tumor cell-derived exosomal miRNA to promote cell-type transformation. Chronic lymphocytic leukemia cell-derived exosomes can be transferred to endothelial cells and MSCs within the tumor microenvironment, and exosomal miRNA and protein can induce an inflammatory phenotype in receiving cells that is similar to the inflammatory CAF phenotype. Therefore, these stromal cells acquire an improved ability to proliferate, migrate, and secrete inflammatory cytokines, generating a more suitable microenvironment for cancer progression (Paggetti et al., 2015).

Mesenchymal stem cell-secreted exosomes are ingested by tumor cells and have a complex effect on tumor evolution. As an example, MSC-derived exosomal miR-16 reduces VEGF expression in tumor cells, and as a consequence, tumor angiogenesis is inhibited, which is the first piece of evidence that MSC-derived exosomal miRNA can remodel the tumor microenvironment (Lee et al., 2013). In addition, exosomes secreted by adipose MSCs can transmit a variety of miRNAs to ovarian cancer cells to regulate a variety of proteins, including apoptotic proteins, cyclins and cytokines and their receptors, and inhibit the development of ovarian cancer (Reza et al., 2016). Wang et al. (2014) found that gastric cancer MSCs secrete exosomal miR-21 that can be internalized by gastric cancer HGC-27 cells to significantly enhance their proliferation and migration capacity. Cancer dormancy is one of the challenges of treatment because cancer cells in the quiescent stage exhibit chemotherapy resistance, with a high possibility of recurrence. Bliss and colleagues found that breast cancer cells (BCCs) could “instigate” MSCs to release specific miRNA (such as miR-222/223)-containing exosomes to promote entry of BCCs into a dormant phase and confer drug resistance. Based on these findings, the authors injected anti-miR-222/223-transfected MSCs in a mouse model to target dormant breast cancer cells, which increased the susceptibility of BCCs to carboplatin and prolonged host survival (Bliss et al., 2016).

In summary Exosomal miRNA works as an intercellular “messenger” to convert the tumor microenvironment into a pro-tumorigenic environment. Donor cells can selectively package and release specific miRNAs to induce changes in biological activities in recipient cells. On the other hand, several interventions, such as chemical agents, can modify the quantity of exosomes released to exert their antitumor effect.

## Endogenous Exosomal miRNA Reprograms the Metastatic Microenvironment

Recent statistical analyses have shown that more than 90% of cancer patients die of tumor metastasis (Marx, 2013). Metastatic tumors have different biological characteristics than primary tumors. When tumor cells migrate to a new site, they often face more challenges, such as attack from the immune system, failure to form abundant blood vessels to maintain nutrition and oxygen supplies, and a stress effect of the oxygen-enriched environment. To enable tumor cells to survive in a new metastatic microenvironment, what Paget (1989) proposed as a “soil microenvironment to be suitable for seed growth,” various cells in the microenvironment must adopt adaptive measures. Tumor cells that have spread to distal organs may retain long-term silence or dormancy when the “soil” environment is not suitable, but the dormant “seed” may form a life-threatening metastasis with progressive adaptation to the “soil” environment, and exosomes are involved in the interaction between the “seed” and “soil.”

Organotropic metastasis is a significant characteristic of some tumors. Evidence has shown that exosomes can direct tumor cells to different target organs. B16-F10 melanoma exosomes injected into mice preferred to transfer to the sentinel lymph nodes, which are close to the injection site. In addition, these exosomes prepared a favorable premetastatic environment to recruit melanoma cells into the exosome-rich sentinel lymph nodes (Hood, San & Wickline, 2011). Moreover, some studies have provided a potential link between selective metastasis and exosomal proteins. Hoshino et al. verified that exosomal integrins can bind to organ-resident cells and mediate organotropic metastasis by activating the expression of pro-migratory genes. For instance, exosomal integrins α_6_β_4_ specifically bind to lung-resident fibroblasts and epithelial cells, directing tumor cell metastasis to the lung, and exosomal integrins α_*v*_β_5_ bind to Kupffer cells, favoring liver tropism, while brain-tropic primary tumor-derived exosomal integrins β_3_ bind brain-resident endothelial cells, inducing brain metastasis (Hoshino et al., 2015). In addition to exosomal proteins, exosomal miRNAs can also reprogram the metastatic microenvironment (Table 1).

**Table 1:** Exosomal miRNA reprograms the metastatic microenvironment.

Donor cells	Recipient cells	microRNA	Targets	Bio-effects	Metastatic sites	Reference
MBCCs	ECs	miR-105	ZO-1	Metastasis↑	Lung and brain	Zhou et al. (2014b)
Brain MBCCs	ECs	miR-181c	PDPK1	Metastasis↑	Brain	Tominaga et al. (2015)
Renal CSCs	ECs	Angiogenic miRNAs	VEGFR1, VEGF MMP2	Metastasis↑	Lung	Grange et al. (2011)
Lung adenocarcinoma	ECs	miR-192	IL-8, ICAM and CXCL1	Metastasis↓	Bone	Valencia et al. (2014)
Breast CCs	Lung fibroblasts, brain astrocytes	miR-122	PKM2, GLUT1	Metastasis↑	Brain and lung	Fong et al. (2015)
Pancreatic adenocarcinoma (rat)	Lymph node SCs and lung fibroblasts	miR-494, miR-542-3p	Cdh17, MAL, TRAF	Metastasis↑	Lymph nodes and lung	Rana, Malinowska & Zoller (2013)
Brain astrocytes	BrMCCs	miR-19a	PTEN	Metastasis↑	Brain	Zhang et al. (2015)
BM-MSCs	MBCCs	miR-23b	MARCKS	Dormant↑	Bone marrow	Ono et al. (2014)

**Notes:**

MBCCs, metastatic breast cancer cells; ECs, endothelial cells; CSCs, cancer stem cells; CCs, cancer cells; SCs, stromal cells; cdh17, cadherin-17; MAL, myelin and lymphocyte protein; TRAF4, TNF receptor-associated factor 4; BrMCCs, brain metastatic cancer cells; BM-MSCs, bone marrow mesenchymal stem cells.

↑: Promoted.

↓: Inhibited.

### Tumor cell-derived exosomal miRNA reprograms the premetastatic niche

For tumor cells to metastasize, it is necessary to improve the permeability of the vascular endothelium and allow circulating tumor cells to reach a premetastatic site. miR-105 is a characteristic miRNA of metastatic breast cancer cells and can be released into the extracellular environment through exosomes. Exosomal miR-105 suppresses expression of the tight junction protein ZO-1 in endothelial cells to destroy vascular endothelial barriers at both primary and secondary sites to promote tumor metastasis. In vivo mouse experiments have shown that overexpression of miR-105 in nonmetastatic tumor cells also increases vascular permeability at metastatic sites and promotes metastasis of breast cancer to the brain and lung (Zhou et al., 2014b). Tominaga et al. reported that to form brain metastases, circulating cancer cells need to first destroy the blood-brain barrier (BBB). miR-181c-containing exosomes from brain-metastatic breast cancer cells can be transferred to endothelial cells of the BBB to destroy the BBB by inhibiting 3-phosphoinositide-dependent protein kinase 1 (PDPK1) expression and altering actin dynamics, which can facilitate brain metastasis of breast cancer cells (Tominaga et al., 2015). MVs released from human kidney cancer stem cells contain angiogenic miRNAs and mRNAs that upregulate expression of VEGF receptor-1 (VEGFR1), VEGF and matrix metallopeptidase 2 (MMP2) in lung endothelial cells and stimulate formation of blood vessels, which contribute to lung metastasis (Grange et al., 2011). In contrast, if the exosomes are enriched with anti-angiogenic miRNA, the exosome-mediated interaction between tumor and stroma cells in the metastatic environment can inhibit tumor metastasis. Valencia et al. have reported that miR-192 overexpressed in lung adenocarcinoma A549 cells can be incorporated into endothelial cells in the osseous microenvironment via exosome-like vesicles. miR-192 impairs angiogenesis and reduces bone colonization of tumor cells by downregulating the expression of proangiogenic intercellular adhesion molecule 1 (ICAM-1), IL-8 and chemokine ligand 1 (CXCL1) (Valencia et al., 2014).

Warburg found that even under aerobic conditions, cancer cells can utilize glycolysis rather than oxidative phosphorylation to produce energy for metabolism, which is believed to be the underlying cause of tumorigenesis and tumor development (Vander Heiden, Cantley & Thompson, 2009; Warburg, 1956). Tumor cells have evolved a variety of ways to increase glucose uptake and utilization. Fong et al. reported that tumor cells can reduce the expression of pyruvate kinase M2 (PKM2) and glucose transporter 1 (GLUT1) by secreting and transferring miR-122-containing extracellular vesicles to stromal cells within the premetastatic niche, including lung fibroblasts and brain astrocytes. Consequently, there is more glucose available for metastatic tumor cells and tumor metastasis is promoted (Fong et al., 2015).

Tumor metastasis is a multistep cascade process that requires cooperation among various factors (Fidler & Poste, 2008). It has been reported that metastatic rat adenocarcinoma BSp73ASML cell-derived exosomes can be absorbed by lymph node stromal cells and lung fibroblasts in vivo after subcutaneous injection. These exosomes contain specific proteins, mRNAs and miRNAs that can affect the expression of cadherin, matrix metalloproteinase, adhesion molecules and pro-angiogenesis factors in target cells in the premetastatic microenvironment to promote tumor metastasis (Rana, Malinowska & Zoller, 2013).

### Stromal cell-derived exosomal miRNA reprograms tumor cells in the metastatic microenvironment

To achieve colonization, metastatic tumor cells in distal organs can be remodeled by stromal cells in the metastatic niche. For example, it has recently been reported that brain-metastatic tumor breast cancer cells can be remolded by astrocyte-derived exosomes in the brain niche. Astrocyte-derived exosomal miR-19a can suppress PTEN expression in brain-metastatic tumor cells, resulting in high chemokine (C–C motif) ligand 2 (CCL2) secretion. IBA1^+^/CCR2^+^ myeloid cells can be recruited to the metastatic microenvironment by CCL2 to support brain metastasis outgrowth by inhibiting apoptosis and promoting proliferation in metastatic breast tumor cells (Zhang et al., 2015).

Tumor cells may enter into a temporary dormant stage after migrating to a new environment. Ono et al. established such a model, and after co-culturing mouse bone marrow-infiltrated breast cancer (BM2) cells with human bone marrow mesenchymal stem cells (BM-MSCs), they found that expression of myristoylated alanine-rich C-kinase substrate(MARCKS), a target gene of miR-23b that is related to cell invasion and cell cycling, was significantly reduced in BM2 cells via transfer of BM-MSC-derived exosomal miR-23b. Thus, breast cancer cells in a metastatic microenvironment exhibit decreased proliferation and migration, with entry into a dormant phase and concomitant reduced sensitivity to many chemotherapeutic agents (Ono et al., 2014).

Through distal transfer, tumor cell-derived exosomal miRNA educates cells in the premetastatic niche, while tumor cells can also be regulated by stromal cell-released exosomal miRNA in the metastatic microenvironment. In other words, exosomal miRNA orchestrates a fertile “soil” for the “seed” to strike root and sprout or provides protective mechanisms against harmful conditions.

## Exogenous Exosomal miRNA Reprograms the Tumor Microenvironment

Previous studies have shown that miRNAs not only are synthesized endogenously but also can be acquired from food and other exogenous routes, such as milk, herbs and microbial infection (Hata et al., 2010; Yang et al., 2015; Zhang et al., 2012; Zhou et al., 2015).

There are large amounts of exosomal miRNAs in bovine milk. These miRNAs are protected from RNase digestion through encapsulation in vesicles and can remain stable under acidic conditions that mimic the gastric intestinal environment (Cui et al., 2017; Hata et al., 2010). Some studies indicate that bovine milk exosomes can be ingested by human macrophages and colon carcinoma cells (Izumi et al., 2015; Wolf, Baier & Zempleni, 2015). One study showed that the miRNAs in cow milk-derived exosomes have specific biological functions to regulate gene expression in human cells (Baier et al., 2014). Breast milk contains abundant immune-related miRNAs, which may be ingested by an infant’s digestive tract via exosome transfer and participate in the development of the immune system (Zhou et al., 2012). A statistical study showed that increased milk consumption may increase the risk of HCC (Duarte-Salles et al., 2014). Milk-derived exosomal miR-155 promoted the expression of STAT3 by downregulating cytokine signaling 1 (SOCS1), a known target for miR-155 (Banikazemi et al., 2017; Cao et al., 2013). As an important mediator in carcinogenesis, STAT3 can upregulate the expression of oncogenic miR-21 to influence the development of tumors (Loffler et al., 2007).

In addition, viral infection is one of the etiological factors for the occurrence of tumors because insertion of a viral genome into a host’s genome may activate proto-oncogenes or inactivate tumor-suppressor genes, destabilizing the host genome (Buchkovich et al., 2008; Elgui de Oliveira, 2007; Fujimuro et al., 2003; Gasser & Raulet, 2006). Through host cells, viruses are capable of synthesizing and releasing miRNA to influence the growth of tumors. For example, Epstein–Barr virus (EBV)-infected nasopharyngeal carcinoma cell-secreted exosomes are enriched with signal transduction molecules, latent membrane protein 1 and virus-encoded miRNAs. These exosomes can be internalized by recipient cells in the tumor microenvironment, such as endothelial cells and fibroblasts, and induce activation of ERK and PI3K/AKT signaling pathways to modulate the growth of neighboring cells (Meckes et al., 2010). Furthermore, exosomes secreted by an EBV-positive gastric cancer cell line transfer miR-BART15-3p into adjacent immune cells, leading to downregulation of the apoptosis inhibitor BRUCE to induce cell apoptosis, engendering a favorable niche for the growth of EBV-infected tumors (Choi et al., 2013). In addition, EBV-infected cells package EBV-encoded BART-miRNA into exosomes that are transferred into noninfected neighboring monocyte-derived dendritic cells (MoDCs), and the EBV-encoded BART-miRNA targets the CXCL11/ITAC gene, indicating that exogenous miRNA introduced via exosomal transfer can function similar to endogenous miRNA in recipient cells (Pegtel et al., 2010).

**Figure 2:**
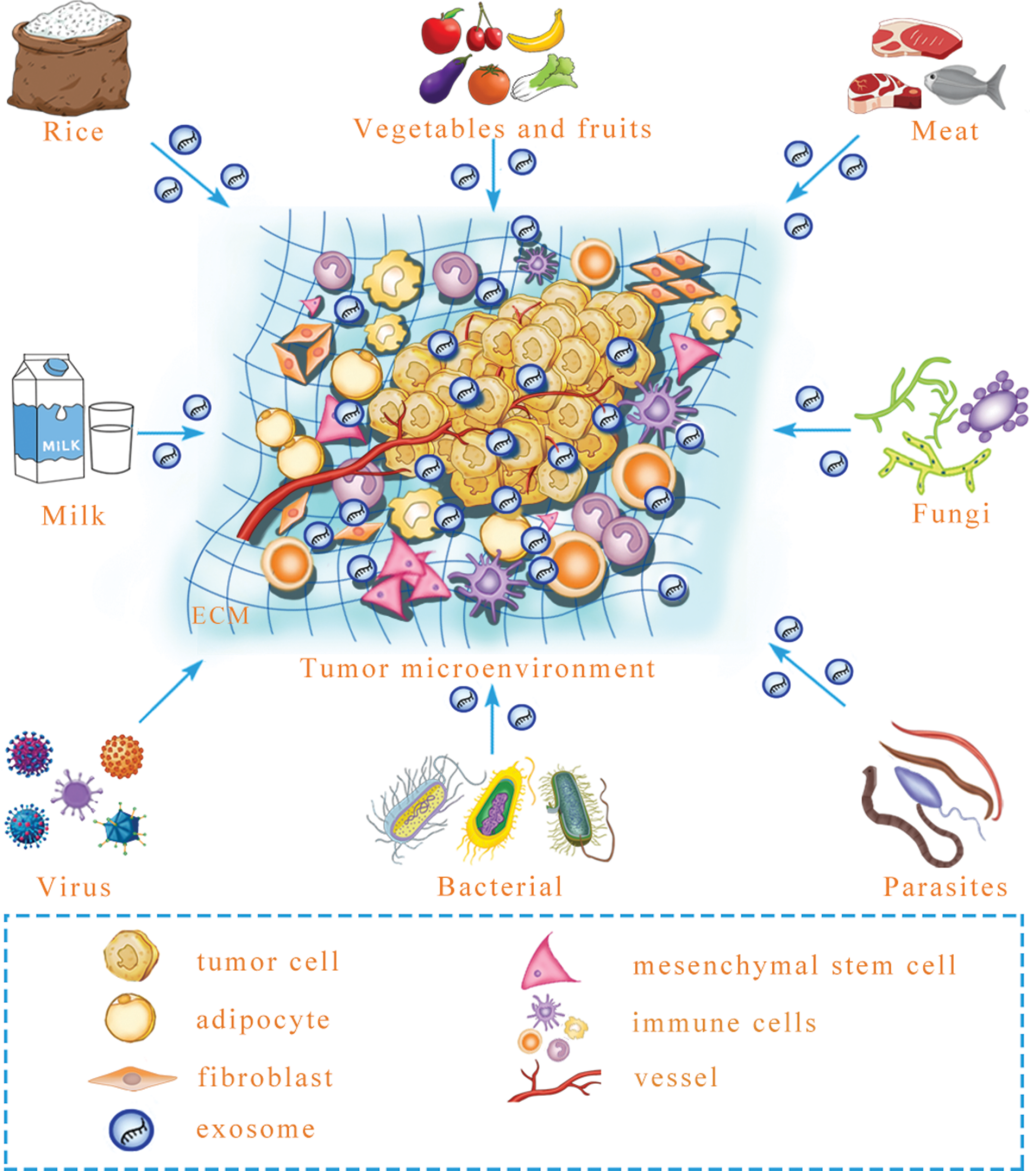
A prediction: various exogenous exosomal miRNAs can reprogram the tumor microenvironment. Certain foods, including rice, meat, vegetables and fruits, may contain exosomal miRNA, and after microbe infection, microbe-derived exosomal miRNA may be released. Whether these exogenous exosomal miRNAs can reprogram the tumor microenvironment is an urgent question that needs to be answered. ECM, extracellular matrix.

All the instances mentioned above suggest that mammalian cells can ingest exogenous exosomal miRNA that regulates target genes. Predictably, exogenous exosomal miRNA can influence intercellular communication to reprogram the tumor microenvironment. Microbial infection can increase the level of exosomal miRNA in the plasma to influence the growth and angiogenesis of tumors in a mouse model (Yang et al., 2017). However, besides virus, whether other microorganisms, including parasites, fungi and bacteria, can secrete exosomes that contain functional miRNA and regulate tumor microenvironment formation is a necessary and urgent issue to be examined (Fig. 2). Similarly, milk-derived exosomes can be used as bioactive carriers to deliver chemotherapeutic drugs, with the advantages of improved bioavailability and efficacy; however, it is unclear whether the construction of antitumor miRNA-containing milk exosomes to target tumors is possible for long-term use (Munagala et al., 2016). In the future, exogenous exosomal miRNA might be remodeled to prevent and target tumors. Although there is research that has yet to be performed in this field, previous studies have provided a new perspective for tumor treatments. In addition to the internal environment, certain interference factors from the external environment also play an important role in the occurrence and progression of tumors. To achieve improved tumor treatment, it is necessary to focus on both internal and external environmental changes to maintain homeostasis of the human body.

## Perspectives and Conclusions

As an important molecule for intercellular communication, an increasing number of studies are focusing on exosomal miRNA-mediated crosstalk among different types of cells in the tumor microenvironment. It is well established that the tumor and its surroundings secrete exosomes that convert the normal tissue microenvironment into a pro-tumorigenic environment through bilateral miRNA exchange. However, the mechanisms by which exosomal miRNA reprograms the tumor microenvironment are not fully understood.

### Selective secretion of exosomal miRNA

As an intercellular signaling molecule, miRNA can exist in many forms: freely, in complex with other proteins or encapsulated in exosome (Arroyo et al., 2011; Vickers et al., 2011). This finding fully demonstrates the flexibility of the biological function of miRNA. Nonetheless, the mechanisms by which different donor cells selectively encapsulate specific miRNA in exosomes have not been elucidated under various conditions, such as hypoxia. Ohshima et al. (2010) analyzed 46 types of tumor cell-derived exosomes, each from a different tissue, and found that AZ-P7a cell lines, which have higher metastatic ability, can produce more exosomes than parental AZ-521 cell lines and that only AZ-P7a cells can selectively secrete tumor suppressive let-7 miRNA-containing exosomes to the extracellular space to maintain high carcinogenic and metastatic capacities. To date, most of the literature shows that this selective secretion is often not dependent on the amount of miRNA in donor cells, and the specific mechanisms need to be clarified.

### Cooperation of exosomal miRNA and other exosome components

miRNA and other exosome components, such as various protein, mRNA, and DNA, have an important role in tumor progression (Hong et al., 2009; Lee et al., 2014; Xiang et al., 2009). However, researchers have paid more attention to the effect of only one component of exosomes, and it is unclear whether different miRNA components or different types of biological molecules in exosomes exert synergistic effects. Skog et al. (2008) provided evidence that glioblastoma cell-derived exosomes containing mRNAs, miRNAs and angiogenic proteins can engender a favorable microenvironment for cancer development and metastatic spread. Thus, further elucidation of the synergistic effect among the different components of exosomes will be beneficial for a comprehensive understanding of the occurrence, progression and metastasis of tumors.

### Exosomes as molecular tumor markers for clinical diagnosis

Exosomes can be released into the blood, urine, saliva, cerebrospinal fluid, synovial fluid, pleural effusion and ascites (Keller et al., 2011; Navabi et al., 2005; Yang et al., 2014). Therefore, exosomes in body fluids can be used as biomarkers of tumor diagnosis and prognosis, and the following questions should be addressed by studies in this field. First, it is well known that exosomal miRNA from tumor cells and the surrounding microenvironment change in both composition and abundance during tumor evolution or after chemotherapy and radiotherapy (Bryant et al., 2012; Gernapudi et al., 2015; Tadokoro et al., 2013; Tang et al., 2016). To better apply exosome detection for clinical diagnosis and treatment, real-time monitoring of exosomal miRNA in the different stages of tumor occurrence, development and treatment should be conducted. Moreover, because cells in both the tumor internal and external environments can release exosomes and because there is no effective method to enrich for different sources of exosomes, the value of the “exosome pool” for clinical diagnosis and treatment still needs to be improved. Finally, evolution of the tumor microenvironment stems from reciprocal crosstalk between tumor cells and stromal cells. In addition to tumor cell-derived exosomal miRNA, stromal cell-derived exosomal miRNA also has significance for clinical diagnosis and treatment.

### Improvement and application of a tumor-related exosomal miRNA database

With the rapid development of microarray and next-generation sequencing technologies, a tumor-related high-throughput database of exosomal miRNA is also accumulating. At present, we can acquire basic information about exosomal miRNA through such websites as ExoCarta (http://www.exocarta.org), Vesiclepedia (http://www.microvesicles.org) and EVpedia (http://evpedia.info) (Kalra et al., 2012; Keerthikumar et al., 2016; Kim et al., 2015). TCGA (http://cancergenome.nih.gov/), Arrayexpress (http://www.ebi.ac.uk/arrayexpress/) and other platforms can also be used to download and apply initial data from exosomal miRNA research (Brazma et al., 2002; Kalra et al., 2012; Keerthikumar et al., 2016; Kim et al., 2015; Zhou et al., 2017). These platforms are convenient tools for scientific researchers. However, compared with mRNA expression profiling, most of which involves cell, tissue and patient specimens, information on exosome and exosomal miRNA remains rare. Furthermore, most studies to date have focused on analyzing tumor cell-derived exosomal miRNA under different in vitro conditions or exosomal miRNA in serum from cancer patients under different clinical intervention modes. This lack of data greatly restricts research progress in the field. For this reason, accumulation and improvement of tumor-related exosomal miRNA databases are imperative.

In addition, due to the insufficiency of pathologic technology for exosome study, it is difficult to quantitate and locate exosomal miRNA in clinical tissue specimens, and accordingly, most of the literature thus far is related to cellular investigation rather than examination of clinical tumor specimens.

In conclusion, the remodeling of the tumor microenvironment is dynamic. Whether constructing a better microenvironment for tumor survival or preparing a suitable metastatic microenvironment, endogenous exosomal miRNA-mediated intercellular crosstalk cross is an important component of the regulation process. However, some evidence has also shown show that exogenous miRNA may also play a pivotal role in remodeling the tumor microenvironment, and thus it is a valuable field that merits further exploration in the future.

## Additional Information and Declarations

### Data Availability

The following information was supplied regarding data availability: This is a literature review and does not have raw data.

## References

[ref-1] Ambros V (2003). MicroRNA pathways in flies and worms: growth, death, fat, stress, and timing. Cell.

[ref-2] Armstrong DA, Green BB, Seigne JD, Schned AR, Marsit CJ (2015). MicroRNA molecular profiling from matched tumor and bio-fluids in bladder cancer. Molecular Cancer.

[ref-3] Arroyo JD, Chevillet JR, Kroh EM, Ruf IK, Pritchard CC, Gibson DF, Mitchell PS, Bennett CF, Pogosova-Agadjanyan EL, Stirewalt DL, Tait JF, Tewari M (2011). Argonaute2 complexes carry a population of circulating microRNAs independent of vesicles in human plasma. Proceedings of the National Academy of Sciences of the United States of America.

[ref-4] Au Yeung CL, Co NN, Tsuruga T, Yeung TL, Kwan SY, Leung CS, Li Y, Lu ES, Kwan K, Wong KK, Schmandt R, Lu KH, Mok SC (2016). Exosomal transfer of stroma-derived miR21 confers paclitaxel resistance in ovarian cancer cells through targeting APAF1. Nature Communications.

[ref-5] Baier SR, Nguyen C, Xie F, Wood JR, Zempleni J (2014). MicroRNAs are absorbed in biologically meaningful amounts from nutritionally relevant doses of cow milk and affect gene expression in peripheral blood mononuclear cells, HEK-293 kidney cell cultures, and mouse livers. Journal of Nutrition.

[ref-6] Banikazemi Z, Haji HA, Mohammadi M, Taheripak G, Iranifar E, Poursadeghiyan M, Moridikia A, Rashidi B, Taghizadeh M, Mirzaei H (2017). Diet and cancer prevention: dietary compounds, dietary microRNAs and dietary exosomes. Journal of Cellular Biochemistry.

[ref-7] Bliss SA, Sinha G, Sandiford OA, Williams LM, Engelberth DJ, Guiro K, Isenalumhe LL, Greco SJ, Ayer S, Bryan M, Kumar R, Ponzio NM, Rameshwar P (2016). Mesenchymal stem cell-derived exosomes stimulate cycling quiescence and early breast cancer dormancy in bone marrow. Cancer Research.

[ref-8] Bovy N, Blomme B, Freres P, Dederen S, Nivelles O, Lion M, Carnet O, Martial JA, Noel A, Thiry M, Jerusalem G, Josse C, Bours V, Tabruyn SP, Struman I (2015). Endothelial exosomes contribute to the antitumor response during breast cancer neoadjuvant chemotherapy via microRNA transfer. Oncotarget.

[ref-9] Brazma A, Sarkans U, Robinson A, Vilo J, Vingron M, Hoheisel J, Fellenberg K (2002). Microarray data representation, annotation and storage. Advances in Biochemical Engineering/Biotechnology.

[ref-10] Bremnes RM, Donnem T, Al-Saad S, Al-Shibli K, Andersen S, Sirera R, Camps C, Marinez I, Busund LT (2011). The role of tumor stroma in cancer progression and prognosis: emphasis on carcinoma-associated fibroblasts and non-small cell lung cancer. Journal of Thoracic Oncology.

[ref-11] Bryant RJ, Pawlowski T, Catto JW, Marsden G, Vessella RL, Rhees B, Kuslich C, Visakorpi T, Hamdy FC (2012). Changes in circulating microRNA levels associated with prostate cancer. British Journal of Cancer.

[ref-12] Buchkovich NJ, Yu Y, Zampieri CA, Alwine JC (2008). The TORrid affairs of viruses: effects of mammalian DNA viruses on the PI3K-Akt-mTOR signalling pathway. Nature Reviews Microbiology.

[ref-13] Calin GA, Croce CM (2006). MicroRNA signatures in human cancers. Nature Reviews Cancer.

[ref-14] Cao Q, Li YY, He WF, Zhang ZZ, Zhou Q, Liu X, Shen Y, Huang TT (2013). Interplay between microRNAs and the STAT3 signaling pathway in human cancers. Physiological Genomics.

[ref-15] Challagundla KB, Wise PM, Neviani P, Chava H, Murtadha M, Xu T, Kennedy R, Ivan C, Zhang X, Vannini I, Fanini F, Amadori D, Calin GA, Hadjidaniel M, Shimada H, Jong A, Seeger RC, Asgharzadeh S, Goldkorn A, Fabbri M (2015). Exosome-mediated transfer of microRNAs within the tumor microenvironment and neuroblastoma resistance to chemotherapy. Journal of the National Cancer Institute.

[ref-16] Choi H, Lee H, Kim SR, Gho YS, Lee SK (2013). Epstein-Barr virus-encoded microRNA BART15-3p promotes cell apoptosis partially by targeting BRUCE. Journal of Virology.

[ref-17] Clement E, Lazar I, Muller C, Nieto L (2017). Obesity and melanoma: could fat be fueling malignancy?. Pigment Cell & Melanoma Research.

[ref-18] Cook J, Hagemann T (2013). Tumour-associated macrophages and cancer. Current Opinion in Pharmacology.

[ref-19] Cui J, Zhou B, Ross SA, Zempleni J (2017). Nutrition, microRNAs, and human health. Advances in Nutrition.

[ref-20] Duarte-Salles T, Fedirko V, Stepien M, Trichopoulou A, Bamia C, Lagiou P, Lukanova A, Trepo E, Overvad K, Tjonneland A, Halkjaer J, Boutron-Ruault MC, Racine A, Cadeau C, Kuhn T, Aleksandrova K, Trichopoulos D, Tsiotas K, Boffetta P, Palli D, Pala V, Tumino R, Sacerdote C, Panico S, Bueno-de-Mesquita HB, Dik VK, Peeters PH, Weiderpass E, Torhild Gram I, Hjartaker A, Ramon Quiros J, Fonseca-Nunes A, Molina-Montes E, Dorronsoro M, Navarro Sanchez C, Barricarte A, Lindkvist B, Sonestedt E, Johansson I, Wennberg M, Khaw KT, Wareham N, Travis RC, Romieu I, Riboli E, Jenab M (2014). Dairy products and risk of hepatocellular carcinoma: the European prospective investigation into cancer and nutrition. International Journal of Cancer.

[ref-21] Elgui de Oliveira D (2007). DNA viruses in human cancer: an integrated overview on fundamental mechanisms of viral carcinogenesis. Cancer Letters.

[ref-22] Fabbri M, Paone A, Calore F, Galli R, Gaudio E, Santhanam R, Lovat F, Fadda P, Mao C, Nuovo GJ, Zanesi N, Crawford M, Ozer GH, Wernicke D, Alder H, Caligiuri MA, Nana-Sinkam P, Perrotti D, Croce CM (2012). MicroRNAs bind to Toll-like receptors to induce prometastatic inflammatory response. Proceedings of the National Academy of Sciences of the United States of America.

[ref-23] Fidler IJ, Poste G (2008). The “seed and soil” hypothesis revisited. Lancet Oncology.

[ref-24] Fong MY, Zhou W, Liu L, Alontaga AY, Chandra M, Ashby J, Chow A, O’Connor ST, Li S, Chin AR, Somlo G, Palomares M, Li Z, Tremblay JR, Tsuyada A, Sun G, Reid MA, Wu X, Swiderski P, Ren X, Shi Y, Kong M, Zhong W, Chen Y, Wang SE (2015). Breast-cancer-secreted miR-122 reprograms glucose metabolism in premetastatic niche to promote metastasis. Nature Cell Biology.

[ref-25] Fujimuro M, Wu FY, ApRhys C, Kajumbula H, Young DB, Hayward GS, Hayward SD (2003). A novel viral mechanism for dysregulation of beta-catenin in Kaposi’s sarcoma-associated herpesvirus latency. Nature Medicine.

[ref-26] Gasser S, Raulet D (2006). The DNA damage response, immunity and cancer. Seminars in Cancer Biology.

[ref-27] Gernapudi R, Yao Y, Zhang Y, Wolfson B, Roy S, Duru N, Eades G, Yang P, Zhou Q (2015). Targeting exosomes from preadipocytes inhibits preadipocyte to cancer stem cell signaling in early-stage breast cancer. Breast Cancer Research and Treatment.

[ref-28] Grange C, Tapparo M, Collino F, Vitillo L, Damasco C, Deregibus MC, Tetta C, Bussolati B, Camussi G (2011). Microvesicles released from human renal cancer stem cells stimulate angiogenesis and formation of lung premetastatic niche. Cancer Research.

[ref-29] Hanahan D, Weinberg RA (2011). Hallmarks of cancer: the next generation. Cell.

[ref-30] Hannafon BN, Carpenter KJ, Berry WL, Janknecht R, Dooley WC, Ding WQ (2015). Exosome-mediated microRNA signaling from breast cancer cells is altered by the anti-angiogenesis agent docosahexaenoic acid (DHA). Molecular Cancer.

[ref-31] Hata T, Murakami K, Nakatani H, Yamamoto Y, Matsuda T, Aoki N (2010). Isolation of bovine milk-derived microvesicles carrying mRNAs and microRNAs. Biochemical and Biophysical Research Communications.

[ref-32] Hong BS, Cho JH, Kim H, Choi EJ, Rho S, Kim J, Kim JH, Choi DS, Kim YK, Hwang D, Gho YS (2009). Colorectal cancer cell-derived microvesicles are enriched in cell cycle-related mRNAs that promote proliferation of endothelial cells. BMC Genomics.

[ref-33] Hood JL, San RS, Wickline SA (2011). Exosomes released by melanoma cells prepare sentinel lymph nodes for tumor metastasis. Cancer Research.

[ref-34] Hoshino A, Costa-Silva B, Shen TL, Rodrigues G, Hashimoto A, Tesic Mark M, Molina H, Kohsaka S, Di Giannatale A, Ceder S, Singh S, Williams C, Soplop N, Uryu K, Pharmer L, King T, Bojmar L, Davies AE, Ararso Y, Zhang T, Zhang H, Hernandez J, Weiss JM, Dumont-Cole VD, Kramer K, Wexler LH, Narendran A, Schwartz GK, Healey JH, Sandstrom P, Labori KJ, Kure EH, Grandgenett PM, Hollingsworth MA, de Sousa M, Kaur S, Jain M, Mallya K, Batra SK, Jarnagin WR, Brady MS, Fodstad O, Muller V, Pantel K, Minn AJ, Bissell MJ, Garcia BA, Kang Y, Rajasekhar VK, Ghajar CM, Matei I, Peinado H, Bromberg J, Lyden D (2015). Tumour exosome integrins determine organotropic metastasis. Nature.

[ref-35] Hu Y, Li D, Wu A, Qiu X, Di W, Huang L, Qiu L (2017). TWEAK-stimulated macrophages inhibit metastasis of epithelial ovarian cancer via exosomal shuttling of microRNA. Cancer Letters.

[ref-36] Iyengar P, Combs TP, Shah SJ, Gouon-Evans V, Pollard JW, Albanese C, Flanagan L, Tenniswood MP, Guha C, Lisanti MP, Pestell RG, Scherer PE (2003). Adipocyte-secreted factors synergistically promote mammary tumorigenesis through induction of anti-apoptotic transcriptional programs and proto-oncogene stabilization. Oncogene.

[ref-37] Izumi H, Tsuda M, Sato Y, Kosaka N, Ochiya T, Iwamoto H, Namba K, Takeda Y (2015). Bovine milk exosomes contain microRNA and mRNA and are taken up by human macrophages. Journal of Dairy Science.

[ref-38] Jang JY, Lee JK, Jeon YK, Kim CW (2013). Exosome derived from epigallocatechin gallate treated breast cancer cells suppresses tumor growth by inhibiting tumor-associated macrophage infiltration and M2 polarization. BMC Cancer.

[ref-39] Josson S, Gururajan M, Sung SY, Hu P, Shao C, Zhau HE, Liu C, Lichterman J, Duan P, Li Q, Rogatko A, Posadas EM, Haga CL, Chung LW (2015). Stromal fibroblast-derived miR-409 promotes epithelial-to-mesenchymal transition and prostate tumorigenesis. Oncogene.

[ref-40] Junttila MR, de Sauvage FJ (2013). Influence of tumour micro-environment heterogeneity on therapeutic response. Nature.

[ref-41] Kalra H, Simpson RJ, Ji H, Aikawa E, Altevogt P, Askenase P, Bond VC, Borras FE, Breakefield X, Budnik V, Buzas E, Camussi G, Clayton A, Cocucci E, Falcon-Perez JM, Gabrielsson S, Gho YS, Gupta D, Harsha HC, Hendrix A, Hill AF, Inal JM, Jenster G, Kramer-Albers EM, Lim SK, Llorente A, Lotvall J, Marcilla A, Mincheva-Nilsson L, Nazarenko I, Nieuwland R, Nolte-’t Hoen EN, Pandey A, Patel T, Piper MG, Pluchino S, Prasad TS, Rajendran L, Raposo G, Record M, Reid GE, Sanchez-Madrid F, Schiffelers RM, Siljander P, Stensballe A, Stoorvogel W, Taylor D, Thery C, Valadi H, van Balkom BW, Vazquez J, Vidal M, Wauben MH, Yanez-Mo M, Zoeller M, Mathivanan S (2012). Vesiclepedia: a compendium for extracellular vesicles with continuous community annotation. PLOS Biology.

[ref-42] Keerthikumar S, Chisanga D, Ariyaratne D, Al Saffar H, Anand S, Zhao K, Samuel M, Pathan M, Jois M, Chilamkurti N, Gangoda L, Mathivanan S (2016). ExoCarta: a web-based compendium of exosomal cargo. Journal of Molecular Biology.

[ref-43] Keller S, Ridinger J, Rupp AK, Janssen JW, Altevogt P (2011). Body fluid derived exosomes as a novel template for clinical diagnostics. Journal of Translational Medicine.

[ref-44] Kim DK, Lee J, Kim SR, Choi DS, Yoon YJ, Kim JH, Go G, Nhung D, Hong K, Jang SC, Kim SH, Park KS, Kim OY, Park HT, Seo JH, Aikawa E, Baj-Krzyworzeka M, van Balkom BW, Belting M, Blanc L, Bond V, Bongiovanni A, Borras FE, Buee L, Buzas EI, Cheng L, Clayton A, Cocucci E, Dela Cruz CS, Desiderio DM, Di Vizio D, Ekstrom K, Falcon-Perez JM, Gardiner C, Giebel B, Greening DW, Gross JC, Gupta D, Hendrix A, Hill AF, Hill MM, Nolte-’t Hoen E, Hwang DW, Inal J, Jagannadham MV, Jayachandran M, Jee YK, Jorgensen M, Kim KP, Kim YK, Kislinger T, Lasser C, Lee DS, Lee H, van Leeuwen J, Lener T, Liu ML, Lotvall J, Marcilla A, Mathivanan S, Moller A, Morhayim J, Mullier F, Nazarenko I, Nieuwland R, Nunes DN, Pang K, Park J, Patel T, Pocsfalvi G, Del Portillo H, Putz U, Ramirez MI, Rodrigues ML, Roh TY, Royo F, Sahoo S, Schiffelers R, Sharma S, Siljander P, Simpson RJ, Soekmadji C, Stahl P, Stensballe A, Stepien E, Tahara H, Trummer A, Valadi H, Vella LJ, Wai SN, Witwer K, Yanez-Mo M, Youn H, Zeidler R, Gho YS (2015). EVpedia: a community web portal for extracellular vesicles research. Bioinformatics.

[ref-45] Kohlhapp FJ, Mitra AK, Lengyel E, Peter ME (2015). MicroRNAs as mediators and communicators between cancer cells and the tumor microenvironment. Oncogene.

[ref-46] Lai RC, Yeo RW, Tan KH, Lim SK (2013). Exosomes for drug delivery—a novel application for the mesenchymal stem cell. Biotechnology Advances.

[ref-47] Lee TH, Chennakrishnaiah S, Audemard E, Montermini L, Meehan B, Rak J (2014). Oncogenic ras-driven cancer cell vesiculation leads to emission of double-stranded DNA capable of interacting with target cells. Biochemical and Biophysical Research Communications.

[ref-48] Lee JK, Park SR, Jung BK, Jeon YK, Lee YS, Kim MK, Kim YG, Jang JY, Kim CW (2013). Exosomes derived from mesenchymal stem cells suppress angiogenesis by down-regulating VEGF expression in breast cancer cells. PLOS ONE.

[ref-49] Li M, Li J, Ding X, He M, Cheng SY (2010). MicroRNA and cancer. AAPS Journal.

[ref-50] Li L, Li C, Wang S, Wang Z, Jiang J, Wang W, Li X, Chen J, Liu K, Li C, Zhu G (2016). Exosomes derived from hypoxic oral squamous cell carcinoma cells deliver miR-21 to normoxic cells to elicit a prometastatic phenotype. Cancer Research.

[ref-51] Liao J, Liu R, Shi YJ, Yin LH, Pu YP (2016). Exosome-shuttling microRNA-21 promotes cell migration and invasion-targeting PDCD4 in esophageal cancer. International Journal of Oncology.

[ref-52] Loffler D, Brocke-Heidrich K, Pfeifer G, Stocsits C, Hackermuller J, Kretzschmar AK, Burger R, Gramatzki M, Blumert C, Bauer K, Cvijic H, Ullmann AK, Stadler PF, Horn F (2007). Interleukin-6 dependent survival of multiple myeloma cells involves the Stat3-mediated induction of microRNA-21 through a highly conserved enhancer. Blood.

[ref-53] Mao L, Li J, Chen WX, Cai YQ, Yu DD, Zhong SL, Zhao JH, Zhou JW, Tang JH (2016). Exosomes decrease sensitivity of breast cancer cells to adriamycin by delivering microRNAs. Tumor Biology.

[ref-54] Marx V (2013). Tracking metastasis and tricking cancer. Nature.

[ref-55] Meckes DG, Shair KH, Marquitz AR, Kung CP, Edwards RH, Raab-Traub N (2010). Human tumor virus utilizes exosomes for intercellular communication. Proceedings of the National Academy of Sciences of the United States of America.

[ref-56] Montecalvo A, Larregina AT, Shufesky WJ, Stolz DB, Sullivan ML, Karlsson JM, Baty CJ, Gibson GA, Erdos G, Wang Z, Milosevic J, Tkacheva OA, Divito SJ, Jordan R, Lyons-Weiler J, Watkins SC, Morelli AE (2012). Mechanism of transfer of functional microRNAs between mouse dendritic cells via exosomes. Blood.

[ref-57] Munagala R, Aqil F, Jeyabalan J, Gupta RC (2016). Bovine milk-derived exosomes for drug delivery. Cancer Letters.

[ref-58] Navabi H, Croston D, Hobot J, Clayton A, Zitvogel L, Jasani B, Bailey-Wood R, Wilson K, Tabi Z, Mason MD, Adams M (2005). Preparation of human ovarian cancer ascites-derived exosomes for a clinical trial. Blood Cells, Molecules, and Diseases.

[ref-59] Neviani P, Fabbri M (2015). Exosomic microRNAs in the tumor microenvironment. Frontiers in Medicine.

[ref-60] Nieman KM, Kenny HA, Penicka CV, Ladanyi A, Buell-Gutbrod R, Zillhardt MR, Romero IL, Carey MS, Mills GB, Hotamisligil GS, Yamada SD, Peter ME, Gwin K, Lengyel E (2011). Adipocytes promote ovarian cancer metastasis and provide energy for rapid tumor growth. Nature Medicine.

[ref-61] Ohshima K, Inoue K, Fujiwara A, Hatakeyama K, Kanto K, Watanabe Y, Muramatsu K, Fukuda Y, Ogura S, Yamaguchi K, Mochizuki T (2010). Let-7 microRNA family is selectively secreted into the extracellular environment via exosomes in a metastatic gastric cancer cell line. PLOS ONE.

[ref-62] Ono M, Kosaka N, Tominaga N, Yoshioka Y, Takeshita F, Takahashi RU, Yoshida M, Tsuda H, Tamura K, Ochiya T (2014). Exosomes from bone marrow mesenchymal stem cells contain a microRNA that promotes dormancy in metastatic breast cancer cells. Science Signaling.

[ref-63] Paget S (1989). The distribution of secondary growths in cancer of the breast. Cancer and Metastasis Reviews.

[ref-64] Paggetti J, Haderk F, Seiffert M, Janji B, Distler U, Ammerlaan W, Kim YJ, Adam J, Lichter P, Solary E, Berchem G, Moussay E (2015). Exosomes released by chronic lymphocytic leukemia cells induce the transition of stromal cells into cancer-associated fibroblasts. Blood.

[ref-65] Pang W, Su J, Wang Y, Feng H, Dai X, Yuan Y, Chen X, Yao W (2015). Pancreatic cancer-secreted miR-155 implicates in the conversion from normal fibroblasts to cancer-associated fibroblasts. Cancer Science.

[ref-66] Parrott JA, Nilsson E, Mosher R, Magrane G, Albertson D, Pinkel D, Gray JW, Skinner MK (2001). Stromal-epithelial interactions in the progression of ovarian cancer: influence and source of tumor stromal cells. Molecular and Cellular Endocrinology.

[ref-67] Pegtel DM, Cosmopoulos K, Thorley-Lawson DA, van Eijndhoven MA, Hopmans ES, Lindenberg JL, de Gruijl TD, Wurdinger T, Middeldorp JM (2010). Functional delivery of viral miRNAs via exosomes. Proceedings of the National Academy of Sciences of the United States of America.

[ref-68] Rana S, Malinowska K, Zoller M (2013). Exosomal tumor microRNA modulates premetastatic organ cells. Neoplasia.

[ref-69] Reis e Sousa C (2006). Dendritic cells in a mature age. Nature Reviews Immunology.

[ref-70] Renehan AG, Zwahlen M, Egger M (2015). Adiposity and cancer risk: new mechanistic insights from epidemiology. Nature Reviews Cancer.

[ref-71] Reza AM, Choi YJ, Yasuda H, Kim JH (2016). Human adipose mesenchymal stem cell-derived exosomal-miRNAs are critical factors for inducing anti-proliferation signalling to A2780 and SKOV-3 ovarian cancer cells. Scientific Reports.

[ref-72] Richards KE, Zeleniak AE, Fishel ML, Wu J, Littlepage LE, Hill R (2017). Cancer-associated fibroblast exosomes regulate survival and proliferation of pancreatic cancer cells. Oncogene.

[ref-73] Singh R, Pochampally R, Watabe K, Lu Z, Mo YY (2014). Exosome-mediated transfer of miR-10b promotes cell invasion in breast cancer. Molecular Cancer.

[ref-74] Skog J, Wurdinger T, van Rijn S, Meijer DH, Gainche L, Sena-Esteves M, Curry WT, Carter BS, Krichevsky AM, Breakefield XO (2008). Glioblastoma microvesicles transport RNA and proteins that promote tumour growth and provide diagnostic biomarkers. Nature Cell Biology.

[ref-75] Tadokoro H, Umezu T, Ohyashiki K, Hirano T, Ohyashiki JH (2013). Exosomes derived from hypoxic leukemia cells enhance tube formation in endothelial cells. Journal of Biological Chemistry.

[ref-76] Tang Y, Cui Y, Li Z, Jiao Z, Zhang Y, He Y, Chen G, Zhou Q, Wang W, Zhou X, Luo J, Zhang S (2016). Radiation-induced miR-208a increases the proliferation and radioresistance by targeting p21 in human lung cancer cells. Journal of Experimental & Clinical Cancer Research.

[ref-77] Tominaga N, Kosaka N, Ono M, Katsuda T, Yoshioka Y, Tamura K, Lotvall J, Nakagama H, Ochiya T (2015). Brain metastatic cancer cells release microRNA-181c-containing extracellular vesicles capable of destructing blood–brain barrier. Nature Communications.

[ref-78] Umezu T, Ohyashiki K, Kuroda M, Ohyashiki JH (2013). Leukemia cell to endothelial cell communication via exosomal miRNAs. Oncogene.

[ref-79] Umezu T, Tadokoro H, Azuma K, Yoshizawa S, Ohyashiki K, Ohyashiki JH (2014). Exosomal miR-135b shed from hypoxic multiple myeloma cells enhances angiogenesis by targeting factor-inhibiting HIF-1. Blood.

[ref-80] Valencia K, Luis-Ravelo D, Bovy N, Anton I, Martinez-Canarias S, Zandueta C, Ormazabal C, Struman I, Tabruyn S, Rebmann V, De Las Rivas J, Guruceaga E, Bandres E, Lecanda F (2014). miRNA cargo within exosome-like vesicle transfer influences metastatic bone colonization. Molecular Oncology.

[ref-81] Vander Heiden MG, Cantley LC, Thompson CB (2009). Understanding the Warburg effect: the metabolic requirements of cell proliferation. Science.

[ref-82] Vickers KC, Palmisano BT, Shoucri BM, Shamburek RD, Remaley AT (2011). MicroRNAs are transported in plasma and delivered to recipient cells by high-density lipoproteins. Nature Cell Biology.

[ref-83] Wang M, Zhao C, Shi H, Zhang B, Zhang L, Zhang X, Wang S, Wu X, Yang T, Huang F, Cai J, Zhu Q, Zhu W, Qian H, Xu W (2014). Deregulated microRNAs in gastric cancer tissue-derived mesenchymal stem cells: novel biomarkers and a mechanism for gastric cancer. British Journal of Cancer.

[ref-84] Warburg O (1956). On the origin of cancer cells. Science.

[ref-85] Wei Y, Li M, Cui S, Wang D, Zhang CY, Zen K, Li L (2016). Shikonin inhibits the proliferation of human breast cancer cells by reducing tumor-derived exosomes. Molecules.

[ref-86] Wolf T, Baier SR, Zempleni J (2015). The intestinal transport of bovine milk exosomes is mediated by endocytosis in human colon carcinoma caco-2 cells and rat small intestinal IEC-6 cells. Journal of Nutrition.

[ref-87] Xiang X, Poliakov A, Liu C, Liu Y, Deng ZB, Wang J, Cheng Z, Shah SV, Wang GJ, Zhang L, Grizzle WE, Mobley J, Zhang HG (2009). Induction of myeloid-derived suppressor cells by tumor exosomes. International Journal of Cancer.

[ref-88] Xing Z, Li D, Yang L, Xi Y, Su X (2014). MicroRNAs and anticancer drugs. Acta Biochimica et Biophysica Sinica.

[ref-89] Yang M, Chen J, Su F, Yu B, Su F, Lin L, Liu Y, Huang JD, Song E (2011). Microvesicles secreted by macrophages shuttle invasion-potentiating microRNAs into breast cancer cells. Molecular Cancer.

[ref-90] Yang J, Farmer LM, Agyekum AA, Hirschi KD (2015). Detection of dietary plant-based small RNAs in animals. Cell Research.

[ref-91] Yang Y, Liu Q, Lu J, Adah D, Yu S, Zhao S, Yao Y, Qin L, Qin L, Chen X (2017). Exosomes from Plasmodium-infected hosts inhibit tumor angiogenesis in a murine Lewis lung cancer model. Oncogenesis.

[ref-92] Yang J, Wei F, Schafer C, Wong DT (2014). Detection of tumor cell-specific mRNA and protein in exosome-like microvesicles from blood and saliva. PLOS ONE.

[ref-93] Ye SB, Li ZL, Luo DH, Huang BJ, Chen YS, Zhang XS, Cui J, Zeng YX, Li J (2014). Tumor-derived exosomes promote tumor progression and T-cell dysfunction through the regulation of enriched exosomal microRNAs in human nasopharyngeal carcinoma. Oncotarget.

[ref-94] Ye SB, Zhang H, Cai TT, Liu YN, Ni JJ, He J, Peng JY, Chen QY, Mo HY, Jun C, Zhang XS, Zeng YX, Li J (2016). Exosomal miR-24-3p impedes T-cell function by targeting FGF11 and serves as a potential prognostic biomarker for nasopharyngeal carcinoma. Journal of Pathology.

[ref-95] Zhang L, Hou D, Chen X, Li D, Zhu L, Zhang Y, Li J, Bian Z, Liang X, Cai X, Yin Y, Wang C, Zhang T, Zhu D, Zhang D, Xu J, Chen Q, Ba Y, Liu J, Wang Q, Chen J, Wang J, Wang M, Zhang Q, Zhang J, Zen K, Zhang CY (2012). Exogenous plant MIR168a specifically targets mammalian LDLRAP1: evidence of cross-kingdom regulation by microRNA. Cell Research.

[ref-96] Zhang Z, Li X, Sun W, Yue S, Yang J, Li J, Ma B, Wang J, Yang X, Pu M, Ruan B, Zhao G, Huang Q, Wang L, Tao K, Dou K (2017). Loss of exosomal miR-320a from cancer-associated fibroblasts contributes to HCC proliferation and metastasis. Cancer Letters.

[ref-97] Zhang L, Zhang S, Yao J, Lowery FJ, Zhang Q, Huang WC, Li P, Li M, Wang X, Zhang C, Wang H, Ellis K, Cheerathodi M, McCarty JH, Palmieri D, Saunus J, Lakhani S, Huang S, Sahin AA, Aldape KD, Steeg PS, Yu D (2015). Microenvironment-induced PTEN loss by exosomal microRNA primes brain metastasis outgrowth. Nature.

[ref-98] Zhou M, Chen J, Zhou L, Chen W, Ding G, Cao L (2014a). Pancreatic cancer derived exosomes regulate the expression of TLR4 in dendritic cells via miR-203. Cellular Immunology.

[ref-99] Zhou W, Fong MY, Min Y, Somlo G, Liu L, Palomares MR, Yu Y, Chow A, O’Connor ST, Chin AR, Yen Y, Wang Y, Marcusson EG, Chu P, Wu J, Wu X, Li AX, Li Z, Gao H, Ren X, Boldin MP, Lin PC, Wang SE (2014b). Cancer-secreted miR-105 destroys vascular endothelial barriers to promote metastasis. Cancer Cell.

[ref-100] Zhou Z, Li X, Liu J, Dong L, Chen Q, Liu J, Kong H, Zhang Q, Qi X, Hou D, Zhang L, Zhang G, Liu Y, Zhang Y, Li J, Wang J, Chen X, Wang H, Zhang J, Chen H, Zen K, Zhang CY (2015). Honeysuckle-encoded atypical microRNA2911 directly targets influenza A viruses. Cell Research.

[ref-101] Zhou Q, Li M, Wang X, Li Q, Wang T, Zhu Q, Zhou X, Wang X, Gao X, Li X (2012). Immune-related microRNAs are abundant in breast milk exosomes. International Journal of Biological Sciences.

[ref-102] Zhou X, Wen W, Zhu J, Huang Z, Zhang L, Zhang H, Qi LW, Shan X, Wang T, Cheng W, Zhu D, Yin Y, Chen Y, Zhu W, Shu Y, Liu P (2017). A six-microRNA signature in plasma was identified as a potential biomarker in diagnosis of esophageal squamous cell carcinoma. Oncotarget.

